# Umbilical vein catheterisation for the family physician working in primary health care

**DOI:** 10.4102/safp.v66i1.5797

**Published:** 2024-01-30

**Authors:** Indiran Govender, Henry I. Okonta, Olukayode Adeleke, Selvandran Rangiah

**Affiliations:** 1 Department Family Medicine and Primary Health Care, School of Medicine, Sefako Makgatho Health Sciences University, Pretoria, South Africa; 2 Department of Family Medicine and Rural Health, Faculty of Health Sciences, Walter Sisulu University, Mthatha, South Africa; 3 Department of Family Medicine, Faculty of Health Sciences, University of KwaZulu-Natal, Durban, South Africa

**Keywords:** vascular access, umbilical vein, resuscitation, informed consent, emergency, air embolism

## Abstract

This is part of a series of articles on vascular access in emergencies. The other two articles were on intra osseous lines and central venous lines. These are critical lifesaving emergency skills for the primary care professional. In this article, we will provide an overview of umbilical vein catheterisation highlighting its importance, the indications, contraindications, techniques, complications and nursing considerations. By familiarising healthcare providers with this procedure, we hope to enhance their knowledge and skills, ultimately leading to improved outcomes in the neonatal population.

## Introduction

Umbilical vein catheterisation is a unique form of central venous access in neonates up to 14 days old.^1,2^ The umbilical venous access route is employed in clinical situations where access through the peripheral veins is unobtainable or inappropriate. It is useful in preterm and critically ill neonates who require frequent blood sampling, continuous monitoring of central venous pressure, venous oxygen saturation, administration of intravenous fluids, medications and parenteral nutrition.

Healthcare providers managing neonates should be skilled in multiple techniques of venous access as inaccessibility through the preferred routes may not always be possible in vulnerable neonates.

## Relevant anatomy

The umbilical cord develops from the yolk sac and allantois during the fifth week of development within which lies two umbilical arteries and one umbilical vein. The umbilical cord provides a pathway for blood transport from the placenta to the foetus and vice versa.

**FIGURE 1:**
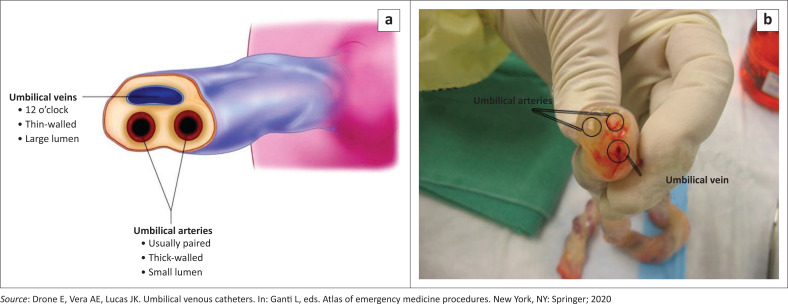
(a) Anatomy of the cord cut transversely about 2 cm from the abdominal wall. (b) Anatomy of the cord cut transversely about 2 cm from the abdominal wall.

The average umbilical cord at birth is 2 cm in diameter and 50 cm long. The arteries have a smaller lumen with thick walls and usually do not bleed because of vasospasm.^3^ The umbilical vein is thin walled with a larger gaping lumen and is usually located at the 12 o’clock position in a cross-section of the umbilical cord (Figure 1).^4^

**FIGURE 2:**
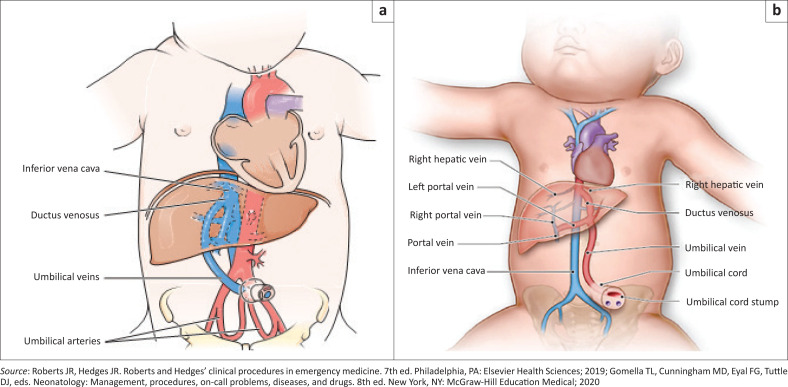
(a) The direct path of an umbilical vein catheter to the inferior vena cava. (b) Anatomic relationships used in the placement of an umbilical venous catheter.

**FIGURE 3:**
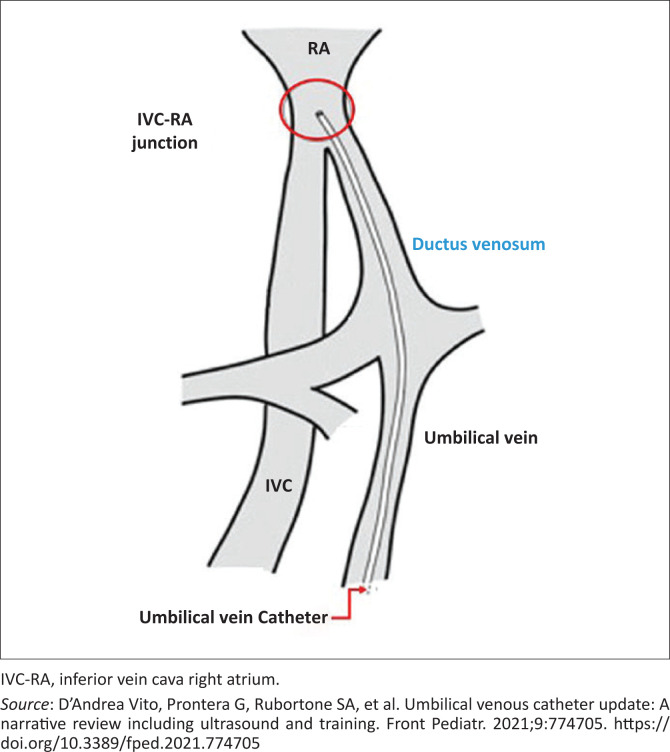
Umbilical vein catheter pathway.

A catheter inserted and advanced in the umbilical vein should normally move from the umbilical vein into the ductus venosus providing direct access to the inferior vena cava (Figure 2 and Figure 3).^3,5^ The relevant anatomic relationships used in the placement of an umbilical catheter is shown in Figure 2b.^6,7^

The umbilical veins arise from a convergence of venules that drain the extra- embryonic allantois.

At the end of 6 weeks, the right umbilical vein is obliterated resulting in a single persisting left umbilical vein allowing for the establishment of two pathways through the liver and heart for transport of oxygenated blood from the placenta to the foetus. At birth the intrabdominal umbilical vein is obliterated producing the ligamentum teres.

## Indications and contraindications

The indications for umbilical venous catheterisation are:

1. Neonates with unobtainable peripheral venous access for emergency resuscitation.^1,3^
2. Exchange blood transfusion.^8^
3. Cardiac catheterisation.^9^

In a quality improvement study, Shahid et al. developed consensus guidelines for the placement of umbilical venous catheters based on gestational age. They recommended the use of umbilical venous catheters in all preterm neonates with a gestational age of ≤ 28 weeks and neonates ≥ 29 weeks gestation who are on mechanical ventilation or have fractional inspired oxygen (FiO_2_) greater than 40% on continuous positive airway pressure or who are haemodynamically unstable.^10^ These recommendations were strategies for reducing unnecessary placements of umbilical venous catheters.

Clinically, this procedure may be used in neonates with respiratory distress syndrome, congenital heart disease, haematological diseases and in neonates requiring surgical procedures, nutritional support, fluid resuscitation and haemodynamic monitoring.

The contraindications for placement of umbilical vein catheters include^1,3^:

1. Gastroschisis
2. Omphalocele
3. Omphalitis
4. Peritonitis
5. Necrotising enterocolitis
6. Air embolism
7. Infection
8. Thrombosis

As infusion through the umbilical catheter can only be started when correct placement is certain, it is important to ensure the availability of a confirmatory imaging facility before this procedure is commenced. Catheters placed too deep can lodge in hepatic vessels where medications may cause hepatocellular damage.

## Equipment required

The equipment for umbilical venous catheterisation include:

1. Imaging facility (preferably ultrasound or thoracoabdominal X-ray).
2. Radiant warmer.
3. Personal protective equipment (sterile gown, sterile gloves, cap, mask).
4. Sterile drape.
5. Disinfectant (chlorhexidine gluconate 1% or povidone-iodine 10%).
6. Curved non-toothed iris forceps.
7. Non-toothed forceps.
8. Scalpel.
9. Scissors.
10. Umbilical tape.
11. 3-O silk suture on a curved needle.
12. Needle holder.
13. Intravenous (IV) tubing and three-way stopcock.
14. Sterile saline flush.
15. Umbilical vein catheter (size 3.5 French or 5 French).

There are polyurethane and polyvinyl umbilical vein catheters that may have single, double or triple lumens. The polyurethane catheters are less prone to bacterial colonisation and are therefore preferred to the polyvinyl catheters.^5^ The use of multiple lumen catheters (double and triple lumen catheters) reduces the need for additional peripherally inserted catheters for simultaneous administration of other infusates or repeated blood samplings. They are, however, more prone to catheter malfunctions.^11^ In resource poor settings where umbilical vein catheters are not available, size 5 feeding tubes can be used as alternatives.^12^

## Preparation

**FIGURE 4:**
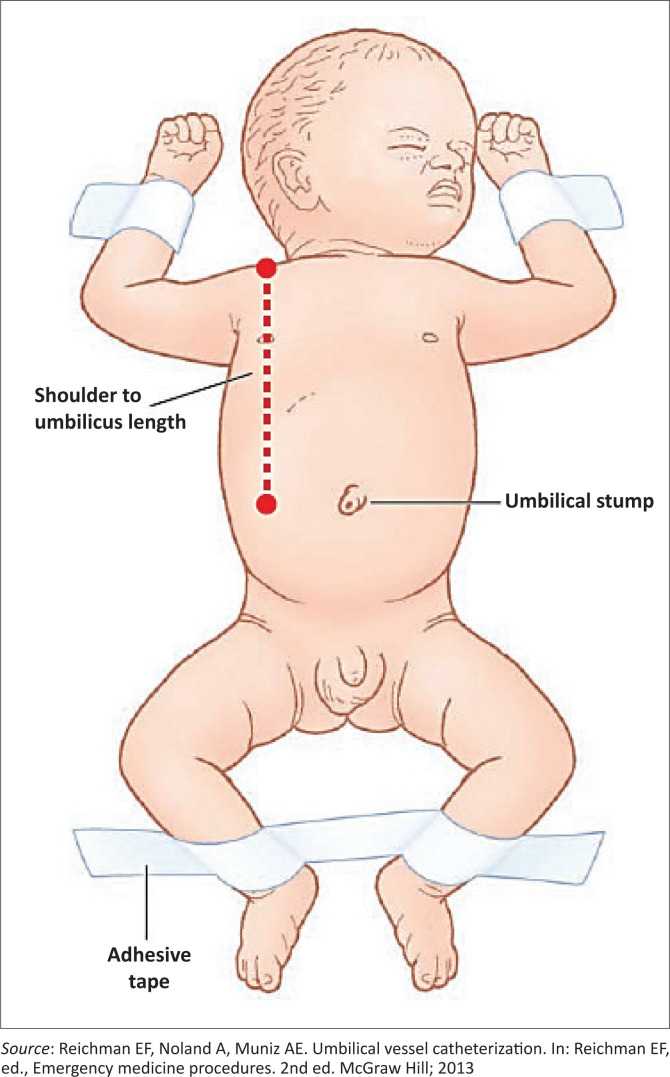
Positioning of the neonate.

Obtain informed consent from the parent or guardian for the procedure. The risks, benefits and potential complications of the procedure should be discussed. In emergency situations where this is not possible, consent is implied. Place the neonate in a cot with a radiant warmer, restrained in a supine position (Figure 4)^15^ and attach the cardiac monitor and pulse oximetry. Select the appropriate catheter size for the body weight and calculate the estimated length to be inserted. The 3.5 French catheters are used for neonates with body weight less than 3.5 kg while the French 5 catheters are used for neonates who weigh 3.5 kg or more.^5,13,14^

Administer 0.1 mL of 33% sucrose solution with a swab orally 2 min before the procedure and repeat the same dose upon commencement of catheterisation.^16,17^ Contraindications to the use of oral sucrose for procedural analgesia include sucrose intolerance, fructose intolerance, glucose intolerance and muscle relaxed neonates and infants.

## Procedure

Put on the sterile protective attire. Flush the catheter with heparinised saline and attach to a closed three-way stopcock, which remains closed till the catheter is in the vein. This is to eliminate air from the catheter and prevent clotting. Clean the umbilical stump and abdomen with disinfectant using an outward spiral motion, and cover with sterile drape that leaves the umbilical area exposed.

**FIGURE 5:**
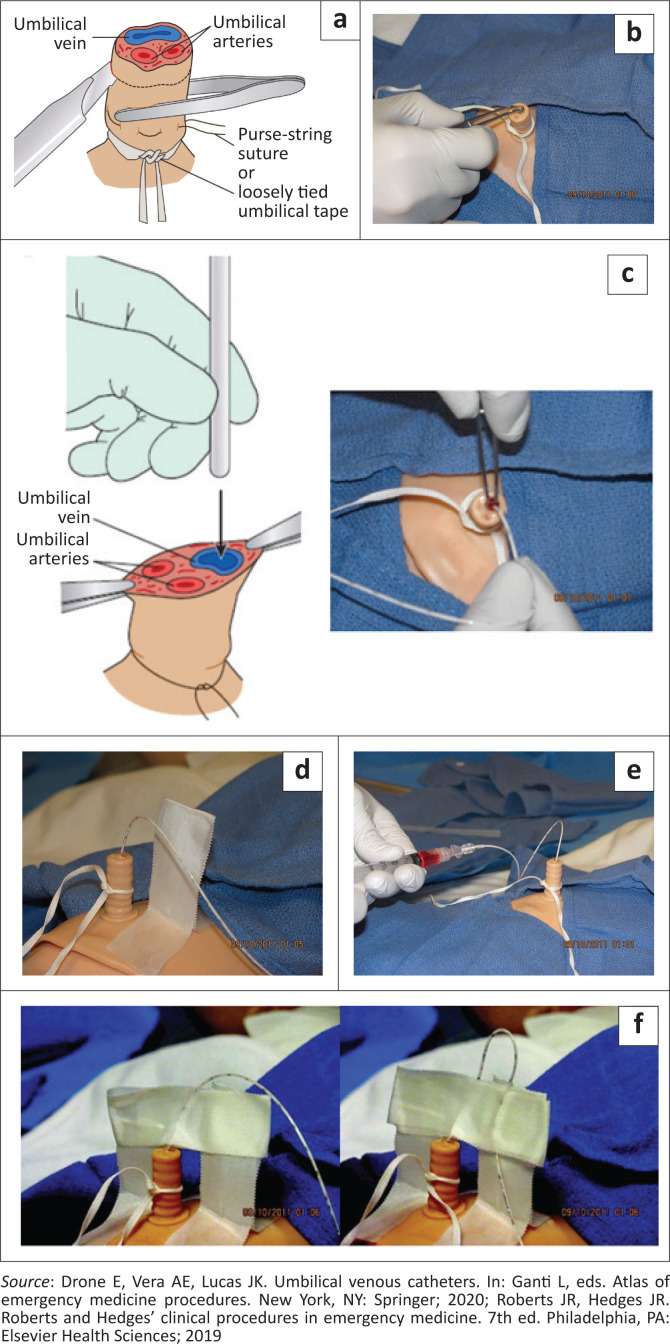
(a–f) Procedure for umbilical vein catheterisation.

Apply a loop of umbilical tape or a purse-string suture with silk at the junction of skin and umbilical stump, secure enough to maintain haemostasis but not too tight to prevent passage of catheter. Make a transverse cut completely through the umbilical stump 1 cm to 2 cm from the skin to expose the non-dried umbilical cord and establish a sterile entry surface (Figure 5a). Identify the umbilical vein, cannulate and dilate it with the iris forceps to clear any thrombus in the lumen (Figure 5b). Holding the base of the umbilical stump with the non-dominant hand, insert and advance the catheter in the umbilical vein, cephalad and to the baby’s right side,^18^ to the desired length (Figure 5c). Some manoeuvres can enhance the passage of the umbilical vein catheter through the ductus venosus. These manoeuvres include manual mobilisation of the liver during the catheter insertion^19^and lying the neonate on the right side to reduce the risk of catheter progression into the portal vein.^20^ If ultrasound machine and expertise is available, compression of the upper abdomen near the portal sinus of the liver to align the umbilical vein and the ductus venosus during catheter insertion can enhance correct placement.^21^ The catheter has markings on it to measure the desired insertion length. Flush the catheter gently only after blood is aspirated freely (Figure 5d).

### Securement

Secure the catheter with a suture through the cord and a tape bridge (Figure 5e). Apply a transparent dressing over the umbilical stump and umbilical catheter (Figure 5f).

**FIGURE 6:**
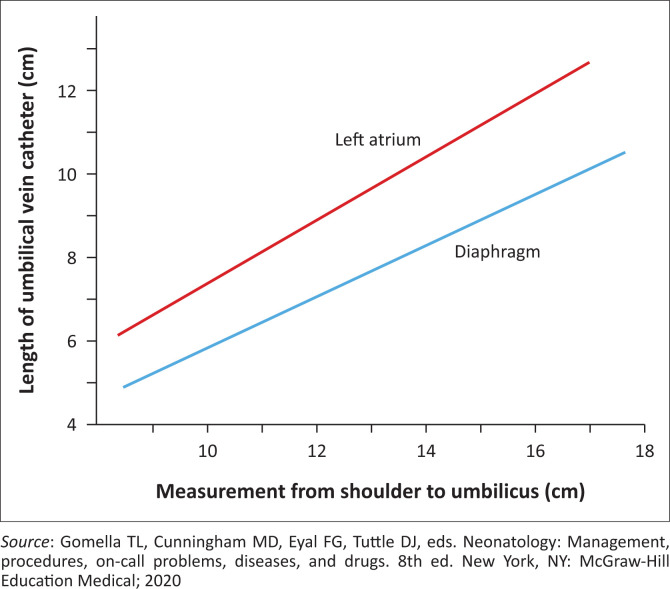
Determining appropriate length of the umbilical venous catheter.

Connect the infusion set to the catheter with no air bubbles in the set. If the catheter placement is for emergency access, advance the catheter 1 cm to 2 cm beyond the point of initial backwash of blood in the catheter, which is usually 4 cm to 5 cm in the full term neonate.^1^ If the line is for long-term access, the catheter should be advanced into the inferior vena cava just beneath the atrium, which is usually 10 cm to 12 cm in the full term neonate.^1^ While using the Dunn method, measure the shoulder-umbilical length, using this length and the desired position of the catheter tip, determine the umbilical catheter length to be inserted into the vein. The length of the umbilical stump should be added to the umbilical vein catheter length found on the y-axis of the graph to determine the corrected length of catheter to be inserted (Figure 6). The catheter length should be between the diaphragm and left atrium on the graph. If resistance is encountered while advancing the catheter, loosen the umbilical tape and attempt to advance the catheter again with gentle pressure.^1^ If significant resistance still persists, do not force the catheter,^3,18^ but abandon the procedure and consider an alternative intravenous access route.^1^

## Catheter tip position confirmation

The catheter tip position should preferably be confirmed with abdominal ultrasonography^5^ or, where this is not available, with a thoracoabdominal x-ray. Current evidence supports the use of ultrasound for localisation of the umbilical vein catheter. It has been suggested as an ideal modality as it accurately confirms the position of the catheter tip and is associated with a decreased radiation exposure than x-ray. The routine use of point-of-care ultrasound (PoCUS) screening at regular intervals in all newborns with a umbilical vein catheter ensures that the catheter is in the proper position and follows any migration of the catheter and detects any complications.^7^ Adequate training is needed prior to the utilisation of PoCUS and achievement of the required competence is feasible following a very brief training period.^22^

The cardiac silhouette and vertebral body methods are used to interpret the thoraco-abdominal x-ray for correct tip position. In the cardiac silhouette method, the curve of the right atrial border is extrapolated medially to its intersection with the inferior vena cava (if catheter is present in it) to determine the target zone, which corresponds to the cavoatrial junction. If the catheter did not pass through the inferior vena cava, the extrapolation is made medially to the right border of the vertebral bodies. In the vertebral body method, the target zone is at the T8–T9 level with the tip lying outside the cardiac silhouette in a lateral X-ray. A well-positioned catheter tip is confirmed if it is in the target zone as estimated by any of the two methods.

The omission of imaging for catheter tip position confirmation may result in failure to detect a catheter tip malposition that carries the risk of morbidities and mortalities from catheter misplacement complications. If the catheter tip position is too high, it should be pulled back to the appropriate position. If the tip position is below the diaphragm, partly withdraw the catheter to the safe short position below the liver or it can be withdrawn completely and a fresh catheter inserted to the appropriate position. Start infusion only when correct placement is confirmed.

It is essential to complete a procedure note in the patient’s file at the end of the umbilical vein catheterisation. The note should include details on the indication for the cathetherisation, date and time of the procedure, means of pain and distress relief, what was performed at every step of the procedure, any complications, estimate of blood loss if any, depth at which the catheter was secured, catheter tip position and how it was confirmed. This procedure note is important for patient follow-up and medico-legal recourse.

## Post-procedure management

A sterile procedure must be used for any manipulation of the umbilical catheter site. The catheter site should be checked every hour,^19^ looking for signs of infection (erythema, warmth, swelling). It must be removed if any of these signs are detected, and the neonate started on broad spectrum antibiotics^19^ while the catheter tip is sent for culture. The site should be kept clean and dry with regular change of dressing. It should also be checked for bleeding and any change in the functioning of the line.

## Complications

While umbilical vein catheterisation is a valuable and commonly performed procedure in neonatal care, it is not without risks and complications. The frequency of these complications is not readily available in the literature although several case reports have been published.^23^

The possible complications of umbilical venous catheterisation include:

1. Vessel perforation and haemorrhage.
2. Infection.
3. Air embolism.
4. Thrombosis.
5. Misplacement into the portal venous system that can lead to hepatic necrosis from injection of hyperosmotic solutions, liver abscess, portal vein thrombosis and cavernoma.
6. Misplacement in the umbilical artery can lead to limb arterial occlusion and limb necrosis.
7. Misplacement into the atrium can lead to perforation, pericardial effusion, cardiac tamponade and cardiac arrhythmias.
8. Catheter migration because of drying of the Wharton jelly and secondary shortening of the umbilical cord. Both inward and outward migration could lead to malposition of the umbilical vein catheter.

### Removal of an umbilical venous catheter

The umbilical vein catheter should be removed as soon as possible to reduce the high risk of colonisation and infection associated with longer indwelling times. Studies suggest limiting umbilical venous catheter dwell time to 7 days,^24,25^ and if prolonged need for a central line is anticipated, the umbilical venous catheter should be replaced with a peripherally inserted central catheter prior to day 4.^26^

To remove the catheter, all infusions must be turned off and ensure that there is no air in the catheter before withdrawal. This is because, if air is present and the infant takes inspiration, the negative pressure generated can pull a significant amount of air into the central vasculature.^4^ The umbilical tie should be loosened, and any suture or tape that would hinder withdrawal should be removed. The catheter should be withdrawn slowly, 1 cm to 2 cm at a time over a few minutes, as a single manoeuvre until about 2 cm to 5 cm remain. The umbilical tie is then tightened while the rest of the catheter is slowly pulled out. Apply gentle pressure above the umbilicus until bleeding has stopped; loosen the umbilical tie.^7^ The tip of the catheter should be sent for culture if infection is suspected. Observe for excessive oozing or haemorrhage.

Preventing or reducing complications associated with umbilical vein catheterisation involves careful attention to procedural techniques, proper maintenance and vigilant monitoring.
